# Active Video Games Training for Older Adults: Comparative Study of User Experience, Workload, Pleasure, and Intensity

**DOI:** 10.2196/67314

**Published:** 2025-05-26

**Authors:** Néva Béraud-Peigné, Alexandra Perrot, Pauline Maillot

**Affiliations:** 1 Université Paris Saclay, Complexité, Innovation et Activités Motrices et Sportives, 335, rue Pierre de Coubertin, Bures-sur-Yvette, 91440, France; 2 Université Paris Cité, Institut des Sciences du Sport-Santé de Paris, Paris, France

**Keywords:** combined training, experience, video game, older adult, elderly, user experience, AVG, UX, physical stimulation, exergame, exergaming, game, gamification, motor, physical, physical activity

## Abstract

**Background:**

Given the appeal of active video games (AVG), many tools are now being used for combined training in older adults. However, there is a lack of comparative data to determine which type of AVG is better suited to older adults.

**Objective:**

The purpose of this study was to compare user experience (UX), workload, pleasure, and intensity of three different experiences: (1) an Immersive and Interactive Wall Exergame (I2WE), (2) a consumer device (SWITCH), and (3) a combination of video games and physical stimulation (biking and videogaming, BIKE-VG) for older adults. I2WE and SWITCH are categorized as Moving While Thinking training, meaning that the cognitive task is integrated into the motor or physical task. In contrast, BIKE-VG is categorized as Thinking While Moving training, where the cognitive and motor or physical tasks are not interconnected. The nature of the cognitive, physical, and motor combinations also differentiates them. I2WE is multi-domain training, while BIKE-VG is physical-cognitive training, and SWITCH is motor-cognitive training.

**Methods:**

A total of 90 older adults (mean [SD] 69.49 [5.78]) were divided into 3 groups (I2WE, SWITCH, and BIKE-VG). Each participant completed a 45-minute group session and then filled out questionnaires to evaluate UX, workload, pleasure, and intensity.

**Results:**

The UX was positive for I2WE and SWITCH, and neutral for BIKE-VG. It was higher for I2WE than for BIKE-VG (*t*_87_=2.83; *P*=.02; *d*=0.70; 95% CI 0.15‐1.69). The workload was moderate across all 3 groups. The intensity was moderate for all groups, ranging between 50% and 70% of the maximum heart rate, and approached high intensity for the I2WE and SWITCH groups. It was significantly higher for I2WE than for BIKE-VG (*t*_66_= 2.86; *P*=.01; *d*=0.70; 95% CI 1.04‐11.43). The perceived pleasure was significantly higher for I2WE (*t*_87_=3.63; *P*=.001; *d*=0.9;95% CI 2.74‐13.23) and SWITCH (*t*_87_=3.11; *P*=.01; *d*=0.87; 95% CI 1.82‐13.69) compared with BIKE-VG.

**Conclusions:**

The UX and perceived enjoyment are higher for the Moving While Thinking training compared with the Thinking While Moving training. This indicates that the I2WE and SWITCH training approaches are promising and motivating options for combined training for older adults.

## Introduction

The interest in training with active video games (AVG) for older adults has been growing over the past few decades [ 1‐2]. AVG are good opportunities to combine cognitive and physical (or motor) stimulation. Moreover, they could increase healthy lifestyle adoption [3], counteract sedentary lifestyles, and enhance individual health behavior, as technology programs provide more enjoyment than traditional interventions [4]. Given the appeal of AVG, many tools are beginning to be used for training, including consumer devices (eg, Xbox, Wii, Switch, and Kinect). The Nintendo Wii, released in 2006, was one of the first consumer devices and was later replaced by the Nintendo Switch (Nintendo Co) in 2017. The Nintendo Wii or Switch console has a wireless handheld pointing device with embedded sensors that detect direction, speed, and acceleration changes in three dimensions. The games offer immediate visual and auditory performance feedback and progress information. More specifically, the cognitive task is incorporated into the motor task by the Moving While Thinking approach [5]. Thus, the 2 tasks are linked and interactive, which could benefit older adults’ cognitive functions since it can increase the synergistic effect of the combined training tenfold [6]. This consumer device allows collective interactions with cooperative and opposition games. In the 2020s, a new type console was developed. These Exergames are assisted by an Immersive and Interactive Wall (I2WE), such as LUMOplay (Lumo Interactive), Play Lü (Lü Interactive Playground) [7], iWall (CSE Entertainment) [8], ExerCube (Sphery AG) [9,10] and Neo One (NeoXperiences) [11,12]. In comparison with consumer devices, I2WE is more extensive and more immersive. I2WE creates simultaneous multi-domain training, with complete physical stimulation (ie, aerobic, muscle strengthening), motor stimulation (complex motor skills), and freedom of movement, in addition to a virtual continuous cognitive stimulation. These characteristics could produce more well-rounded and intensive training than consumer devices.

Other experiences were created with a combination of physical exercise (eg, cycling, kayaking) and virtual stimulation (eg, video games, interactive maps) [13-15]. Cognitive stimulation is sometimes linked (Moving While Thinking, eg, cycling with an interactive map), but other times, they are not (Thinking While Moving, eg, cycling with unrelated video games).

Alongside developing these various tools, many studies have been undertaken to measure their health effects on older adults, including physical [1,16], dual-task [12], cognitive [2,17], and social benefits [18]. However, the positive impacts of AVG on individual cognitive domains are inconsistent across studies [19]. This may result from devices’ varying forms and definitions of AVG’s physical and cognitive demands. Indeed, AVG could generate different solicitations like physical-cognitive training, motor-cognitive training, and multi-domain training [20]. Torre and Temprado [20] described physical-cognitive training as the sequential or simultaneous combination of endurance (aerobic) and muscular resistance exercises with cognitive tasks. Motor-cognitive training, on the other hand, involves pairing complex motor skills training with cognitive tasks that are typically added separately from the motor task. However, we propose that motor-cognitive training can also involve simultaneous motor and cognitive stimulation (eg, consumer device like Nintendo Switch). Multi-domain training integrates aerobic exercises and muscular resistance, complex motor skills, and cognitive tasks. Moreover, the interaction, immersion, controller (ie, body controller, object controller, button controller, mixed button, and gesture controller), and games may differ. As Li et al [18] recommended in their systematic review, more studies are needed to compare the effects of different exergaming platforms. Herold et al [21] indicated that future studies should directly compare the effectiveness of various digital and home-based physical training interventions.

It is highly recommended that AVG for public health and disease prevention be designed to meet the expectations and needs of its target users [22]. Before conducting full-scale studies, Campbell [23] indicated that the acceptability and feasibility should be tested with older adults. The workload must be appropriate to provide sufficient stimulation without causing excessive stress. The same goes for the intensity, which should be moderate according to recommendations [19]. More specifically, to make AVG appealing to older adults, it needs to be designed to encourage initial acceptance and sustained use over time. This requires a user-centered design approach, including thoroughly evaluating the exergames’ user experience (UX) [24]. UX, which results from the interaction between the user, the system, and the context, is crucial in ensuring high-quality user-system interaction. Consequently, UX is increasingly used to assess AVG for the elderly [24-27]. The concept of UX builds upon the foundations of usability, which alone are now seen as inadequate for predicting and explaining technology acceptance. UX expands usability by incorporating emotional, subjective, and temporal dimensions, defining an individual’s technology interaction. These aspects are crucial for designing suitable interactive systems. Hassenzahl [28] uses a pragmatic–hedonic model of UX, in which individuals perceive the quality of their interactions with products according to two types of attributes: pragmatic qualities, directly related to task accomplishment and product use, and hedonic qualities, which emphasize the psychological well-being of individuals. These 2 attributes combine to generate a global evaluation of product attractiveness. In actual studies that evaluated active video game UX among older adults, UX is positive [29,30]. Perceived enjoyment is also often evaluated to complete the UX assessment [31-33]. More enjoyable AVG can lead to more intense activity [34,35].

Numerous studies have been conducted on AVG involving older adults, but few focus on comparing different experiences with each other. Studies have mainly focused on single gaming platforms or compared 2 modes with short game exposures [26] or have lacked participants [30].

Studies agree that AVG creates positive training experiences. However, there is a lack of comparative information to determine whether one type of AVG would be more beneficial than others. The context of this study was primarily focused on a preventive approach, aimed at reducing the risk of physical and cognitive decline associated with aging. The goal was to explore how innovative strategies, such as the use of active video games, could be used as a primary prevention strategy to preserve cognitive function before the onset of impairments. The purpose of this study was to compare three different experiences: (1) I2WE, (2) consumer device (SWITCH), and (3) a combination of video games and physical stimulation (biking and gaming, BIKE-VG). We expected the 3 AVG training sessions create a physical intensity in line with the recommendations for older adults (moderate to high), with a correct session workload and a positive UX. We hypothesized that I2WE and SWITCH would generate a greater UX and intensity than BIKE-BG. Moreover, a higher workload was expected for the Thinking While Moving training (ie, BIKE-VG) compared to the 2 Moving While Thinking trainings (ie, I2WE, SWITCH).

## Methods

### Ethical Considerations

This study was approved by the Ethics and Research Committee of Université Paris-Saclay (CER-Paris-Saclay-2023‐084). Informed consent was obtained from all participants prior to their inclusion in the study. Participants were clearly informed of their right to withdraw from the study at any time without consequence. All data collected during the study were fully anonymized. No financial compensation was provided to participants for their involvement in the study.

### Recruitment

Potential participants responded positively to a call for participation by email or flyer distributed at associations and clubs in Ile-de-France (France). A power analysis using the G*Power version 3.1.9.7 (Heinrich-Heine-University), based on the primary statistical test planned for this study (ANOVA: Fixed effects, omnibus, one-way), revealed that a sample size of 66 participants was needed (*α*=.05; power=0.80; effect size: *f*=0.40). All participants met the following criteria: (1) be over 60 years of age, (2) score three or higher on a subjective health scale (1=verypoor-5=excellent), (3) have no major pathology, and (4) have normal or corrected vision and hearing.

### Protocol

**Figure 1.**
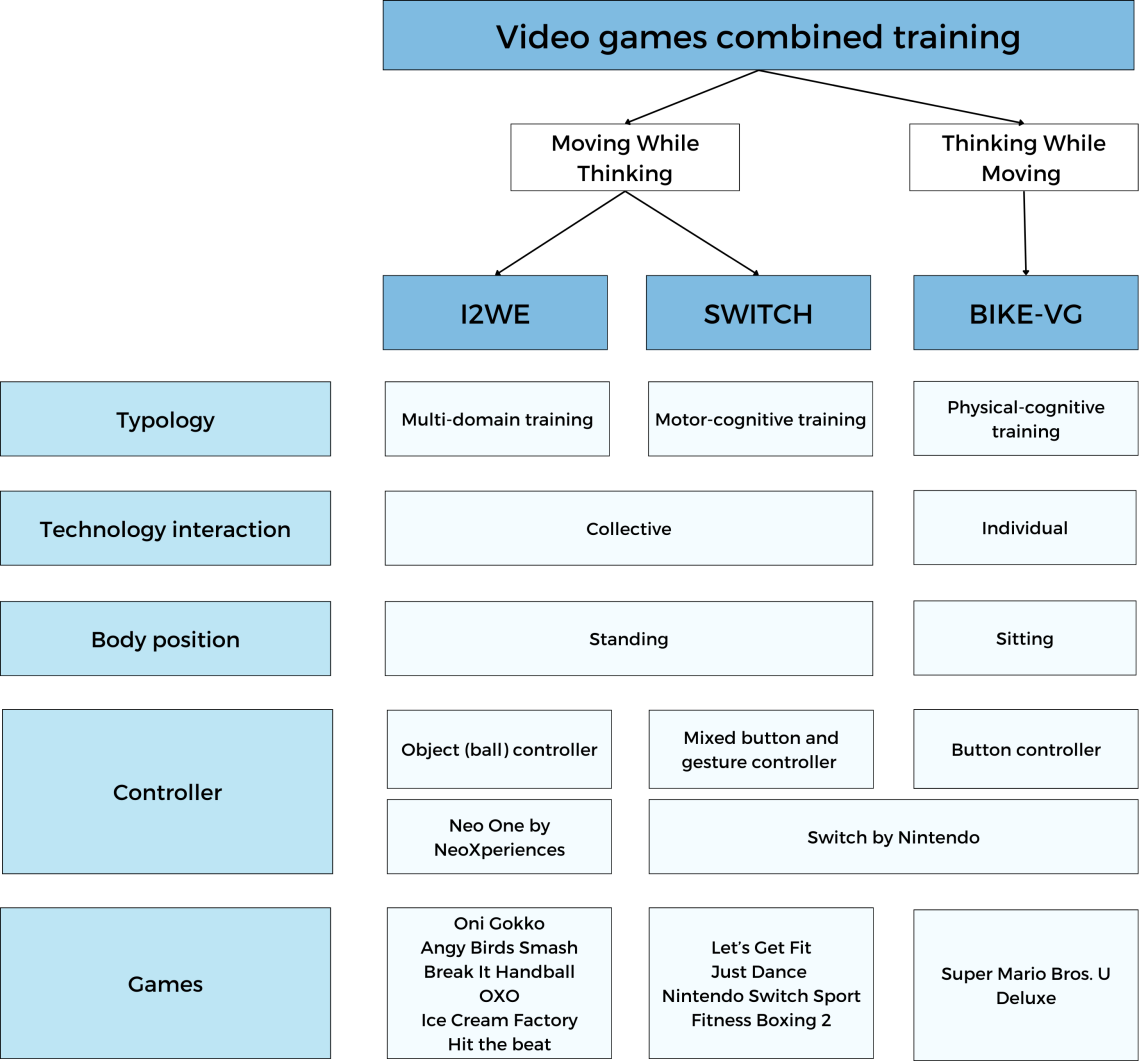
Description of the 3 video games combined training Notes. Three types of video game combined training were compared: (A) Immersive and Interactive Wall Exergame, (B) SWITCH games, and (C) Bike-based Video Games group. I2WE and SWITCH were classified as Moving While Thinking training, meaning that the cognitive task is integrated into the motor/physical task. BIKE-VG was considered Thinking While Moving training, where the cognitive and motor/physical tasks were not interconnected [5]. To define the different types of combined training, we followed the modified definition by Torre and Temprado [20]. I2WE was categorized as multi-domain training (integrates aerobic and muscular resistance exercises, complex motor skills, and cognitive tasks). SWITCH was a motor-cognitive training that paired complex motor skills training with cognitive tasks. BIKE-VG was defined as physical-cognitive training, which involved the combination of endurance and muscular resistance training with cognitive tasks.

Participants were split into 3 groups (ie, I2WE, SWITCH, and BIKE-VG). The characteristics (ie, physical/motor stimulation, interaction, body position, controller, and games) are described in Figure 1.

For each group, the protocol was the same: the participants were divided into groups of four participants per session (45 min) and then filled in a battery of questionnaires. The session was always preceded by ten minutes of exercises, during which the participants gradually warmed up (joint mobilization – eg, turning the head, shoulders, and ankles – walking around the room at various paces, and squat movements). A sports therapist supervised the session. The supervisor ensured participants’ safety by monitoring correct posture. For I2WE and SWITCH, the breaks between the games lasted a few seconds, just enough time to switch to the next game.

#### I2WE Group

**Figure 2.**
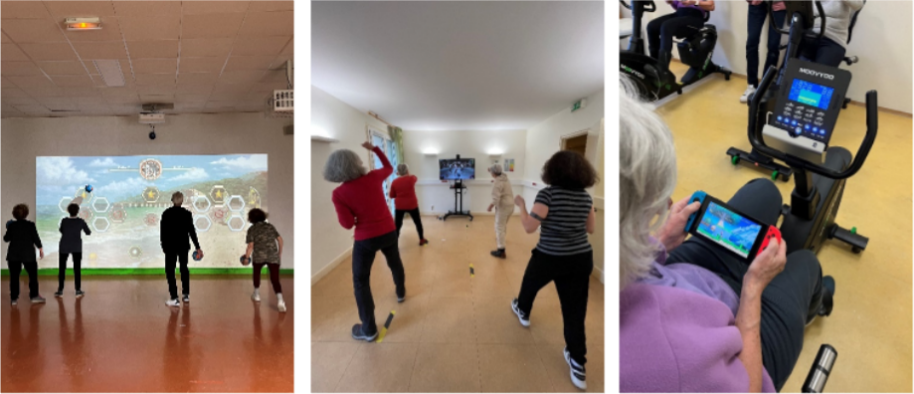
Photography of each group (A) Immersive and Interactive Wall Exergames, (B) SWITCH games, (C) Bike-based Video Games group .

A Neo One device was used (Figure 2A), an I2WE equipped with a video projector and infrared sensors that allow playing without headphones or equipment, so the body controls games. Neo One was conducted standing, offering multi-domain training with collective interactions (ie, cooperation, opposition) between players (multiplayer). Neo One has about 20 games, including sports and puzzle games, that can be played in cooperation or opposition. For this study, the session was composed of 6 games of 5 minutes. The instructions and rules of the games were displayed on the screen and re-explained by the supervisor if necessary. The first 3 games were cooperative, while the next 2 were competitive (2 vs 2). The final game was also competitive but played 1 versus 1.

##### Oni Gokko

Participants had to complete several mini games, including Hangman, Hidden Object, Memory, and Word Scramble. The goal was to score as many points as possible.

##### Angry Birds Smash

In this game, players had to destroy as many fortresses and pigs on the screen as possible to progress through levels. The objective was to complete the levels and achieve the highest score within the allotted time.

##### Break It Handball

In this game, players aimed at various bricks displayed on the screen to advance through levels. The objective was to complete the levels and achieve the highest score within the time limit.

##### OXO

This puzzle game was based on the mechanics of tic-tac-toe. The goal was to align three symbols on the grid to win.

##### Ice Cream Factory

In this game, 2 teams competed to fulfill ice cream orders as quickly as possible and earned coins. The team with the most coins at the end of the time won.

##### Hit the Beat

In this game, participants had to hit targets at the right moment as they appeared in rhythm with the music.

### SWITCH Group

The Nintendo Switch device was a motion-sensitive controller to detect three dimensions (Figure 2B). It was an exergame connected with a 35-inch screen. The controller has mixed buttons and gestures. Exergames were conducted standing, offering motor-cognitive solicitation, with collective interactions (ie, cooperation and opposition) – multiplayer. A total of 5 games of 5‐7 minutes were used. For the first two games, participants played all 4 together. For the last three games, participants were separated into 2 groups of two but played simultaneously.

#### Let’s Get Fit

This game consisted of following the movements of an avatar and performing sports movements (eg, high knees, heel kicks, and squats).

#### Just Dance

The players followed dance choreographies on screen by mimicking the movements of virtual dancers as best as possible. Around 2 pieces of music were played: “*Rain Over Me” and “Le Bal Masqué.”*

#### Football on Nintendo Switch Sport

In this game, 2 participants competed against each other. The aim was to score more goals than their opponent. Each participant moved their avatar with the Joy-Stick and shot the ball with a Joy-Con placed on their leg.

#### Badminton on Nintendo Switch Sport

In this game, 2 players competed against each other. The game used motion controls and button inputs to simulate swings and shots. The aim was to score more points than their opponent.

#### Fitness Boxing 2

It combined boxing-inspired workouts with music and rhythm gameplay elements. Players used the Joy-Con controllers to simulate a series of boxing movements (ie, forehand, hook, uppercut) in time with the music and on-screen prompts.

### BIKE-VG Group

Participants had to play the *Super Mario Bros.U Deluxe* game on the Nintendo Switch while pedaling on a stationary bike (Ultra Green R 2.0 by MOOVYOO), offering physical-cognitive training (Figure 2C). Each participant played individually (single-player). Joy-Con controllers were attached to the console in the participant’s hands, so the controller was only by button. The game involved navigating through side-scrolling levels filled with enemies, obstacles, and secrets. After explaining how to use the game console and the game instructions, a 5-minute warm-up at minimum resistance was performed on the bike. Next, participants had to pedal and play for 40 minutes, with resistance incrementing every 3 minutes to a minimum level of 4. If participants wanted to put on more resistance, they were free to do so.

### Outcomes

#### Heart Rate

The participant’s heart rate was continuously monitored throughout the session with a Polar Verity Sense heart rate monitor. Theoretical Maximum Heart Rate (TMHR) was determined using the formula developed by Gellish and colleagues [36]: TMHR =192‐0.007x age². The subsequent calculation defines the average intensity experienced by the individual during the session: average intensity = (average heart rate x 100) / TMHR.

#### Questionnaires

QAPPA (in French : Questionnaire d’Activité Physique pour les Personnes Agées – in English: Physical Activity Questionnaire for Older Adults) [37] was a questionnaire developed to assess weekly physical activity levels, tailored specifically for older adults.

AttrakDiff 2 [38] which assessed user experience (UX), was translated and validated in French [39]. The questionnaire items were categorized into four scales: (1) Pragmatic Qualities, which evaluated usability and perceived ease of use; (2) Hedonic Quality – Stimulation, which assessed the support for stimulation with new, attractive, and stimulating content, features, and interaction styles; (3) Hedonic Quality – Identity, which evaluated the support for social function and communication of user identity; and (4) Overall Attractiveness, which gauged the overall perceived value of the product based on its pragmatic and hedonic qualities. For each scale, the score ranges from −3 (low score) and 3 (high score).

NASA-Task Load index (NASA-TLX) [40] assessed the workload associated with tasks. Participants rated six dimensions of workload (ie, mental demand, physical demand, time pressure, performance, effort, and frustration) on a scale from 0 to 100. Due to the multidimensional nature of NASA-TLX, some dimensions may contribute more importantly to the total score, prompting weighting procedures to assign coefficients to each dimension. However, these procedures were criticized for being time-consuming [41]. Alternatively, Bittner et al [42] proposed a more straightforward approach by averaging the scores across all six dimensions, termed the “Raw” Task Load Index (RTLX). They demonstrated a high correlation between the RTLX and the traditional calculated score. Cegarra and Morgado [43] confirmed this finding with the French version of the scale.

The physical activity enjoyment scale [44]*,* consisting of ten items translated and validated in French [45] on a scale ranging from 7 to 70 (best score) assessed the perceived enjoyment experienced during physical exercise.

The modified Borg Scale [46] gauged perceived exertion during exercise. It used a numerical scale ranging from 0 to 10, with corresponding verbal anchors (eg, very light, difficult, painful effort) to help individuals rated their exertion level.

#### Want to Continue to Play Active Video Games

Participants were asked if they would like to continue playing AVG in the future (strongly agreed, agreed, don’t know, disagreed, strongly disagreed).

### Statistical Analysis

The analyses were performed using JASP software (0.18.3 version). Descriptive data (ie, mean, SD) were reported for all variables. Personal variables group differences were compared with a one-way analysis of variance (ANOVA) for continuous variables (ie, age, BMI, QAPPA) and *χ*^2^ test for categorical variables (ie, sex, educational level, active video games practice). To determine if the UX for each AVG was positive, a one-sample Student *t* test was used to compare the mean of each sample to a reference value. ANOVAs were employed to compare perceived enjoyment, session workload, physical intensity, and UX between groups. 95% CI completed them for the mean difference. All statistical analyses with a *P* value <.05 were considered significant. A Tukey Post Hoc test was realized when a significant interaction was found. Effect Sizes were calculated to study the power of the results: Eta-Squared (η²) for ANOVA and Cohen *d* for *t* test. As indicated in the effect size guidelines from Cohen [47], η² is considered low if the effect size is between 0.01 and 0.06, medium if it is between 0.06 and 0.14, and large if it is superior to 0.14. Cohen *d* is considered low if the effect size is between 0.20 and 0.50, medium between 0.50 and 0.80, and large if superior to 0.80.

## Results

### Overview

Personal information for each group is given in Table 1. There were no significant differences for all variables.

**Table 1.** Personal variables.

Personal variables	Switch (n=23)	I2WE^a^ (n=38)	Bike-vg^b^ (n=29)	*P* value
Age (years), mean (SD)	69.39 (6.42)	68.26 (5.53)	71.17 (5.34)	.12
BMI (score)	25.09 (4.94)	24.08 (3.33)	24.45 (2.59)	.57
QAPPA^c^ (n)	877.39 (712.01)	1008.95 (683.62)	857.93 (705.43)	.63
Sex, n (%)				.58
Male	4 (17.39)	9 (23.68)	4 (13.79)	
Female	19 (82.61)	29 (76.32)	25 (86.21)	
Level of education, n (%)				.10
No diploma	0 (0)	4 (10.53)	0 (0)	
Certificate	2 (8.7)	5 (13.16)	1 (3.45)	
Vocational certificate	7 (30.43)	12 (31.58)	3 (10.34)	
High school diploma	2 (8.7)	1 (2.63)	3 (10.34)	
Associate’s degree	4 (17.39)	8 (21.05)	5 (17.24)	
Bachelor’s degree	4 (17.39)	7 (18.42)	8 (27.59)	
Master’s degree	4 (17.39)	1 (2.63)	7 (24.14)	
Doctorate degree	0 (0)	0 (0)	2 (6.9)	
Active video games practice (%)				.74
More than one a week	0 (0)	0 (0)	0 (0)	
Between once a week and once a month	0 (0)	1 (2.63)	0 (0)
Between one a month and once a year	3 (13.04)	4 (10.53)	2 (6.90)	
Never	20 (86.96)	33 (86,84)	27 (93.10)	

a I2WE: Immersive and Interactive Wall Exergame

b BIKE-VG: Bike-based Video Game group

c QAPPA: Questionnaire d'Activité Physique Pour les Personnes Âgées (Physical Activity Questionnaire for Older Adults)

### User Experience

**Figure 3.**
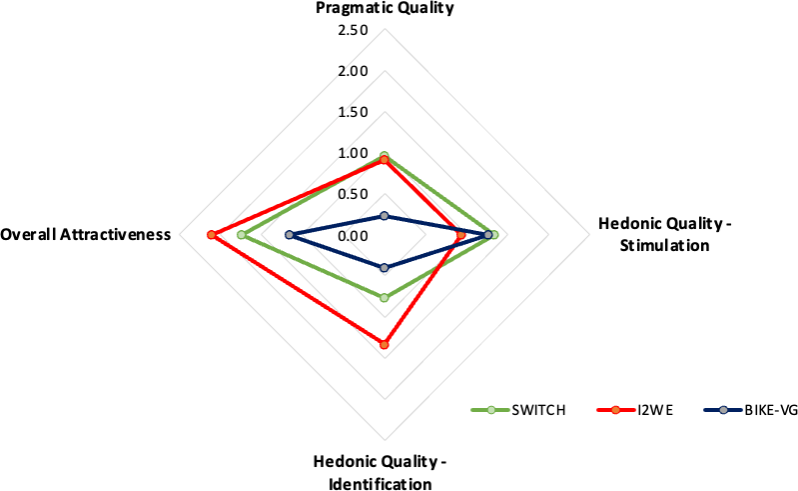
User experience depending on groups.

The UX, depending on the group, are given in Figure 3.

#### Comparison at the Threshold

The area of positivity of the AttrakDiff2 items is between 1 and 3, neutrality between −1 and 1, and negativity between −1 and −3. To know if the scores were significantly positive, a *t* test was performed to compare the averages for the four AttrakDiff2 items with a value of 1 for each group (Table 2).

**Table 2.** Score difference from standard 1.

Score	Mean (SD)		*t* test	*P* value	*d*
I2WE^a^					
Pragmatic quality (score)	0.91 (0.89)		0.65	.52	0.10
Hedonic quality stimulation (score)	1.68 (0.81)		5.18	<.001	0.84
Hedonic quality identity(score)	1.32 (0.69)		2.90	.01	0.47
Total attractiveness (score)	2.08 (0.79)		8.44	<.001	1.37
SWITCH					
Pragmatic quality (score)	0.96 (0.79)		0.22	.82	0.05
Hedonic quality stimulation (score)	1.53 (1.01)		2.49	.02	0.52
Hedonic quality identity (score)	0.91 (0.89)		0.47	.65	0.10
Total attractiveness (score)	1.93 (1.17)		3.78	.001	0.79
BIKE-VG^b^					
Pragmatic quality (score)	0.23 (1.09)		3.79	<.001	0.70
Hedonic quality stimulation (score)	1.26 (0.92)		1.52	.14	0.28
Hedonic quality identity (score)	0.40 (1.22)		2.66	.01	0.49
Total attractiveness (score)	1.16 (1.85)		0.47	.64	0.09

a I2WE: Immersive and Interactive Wall Exergame

b BIKE-VG: Bike-based Video Game group

The Pragmatic Quality is significantly smaller than 1 for BIKE-VG (*t*_28_=3.79; *P*<.001; *d*=0.70). There was no significant difference to 1 for I2WE and SWITCH.

The Hedonic Quality - Stimulation is significantly greater than 1 for I2WE (*t*_37_=5.18; *P*<.001; *d*=0.84) and SWITCH (*t*_22_=2.49; *P*=.02; *d*=0.52).

The Hedonic Quality - Identity is significantly greater than 1 for I2WE (*t*_37_=2.90; *P*=.01; *d*=0.47) and significantly lesser than 1 for BIKE-VG (*t*_28_=2.66; *P*=.01; *d*=0.49).

The Total Attractiveness is significantly greater than 1 for I2WE (*t*_37_=8.44; *P*<.001; *d*=1.37) and SWITCH (*t*_22_=3.78; *P*=.001; *d*=0.79).

### Comparison Between Groups

ANOVA analysis revealed a significant group effect for three items: Pragmatic Quality (*F*_2,87_=5.45; *P*=.01; η²=.11), Hedonic Quality Identity (*F*_2,87_=780; *P*<.001; η²=0.16), and Total Attractiveness (*F*_2,87_=4.31; *P*=.02; η²=.09). Post Hoc analysis indicated that for Pragmatic Quality, the score is significantly better for SWITCH than BIKE-VG (*t*_87_=2.79; *P*=.02; *d*=0.78; 95% CI 0.11‐1.36), and for I2WE than BIKE-VG (*t*_87_=2.92; *P*=.01; *d*=0.72; 95% CI 0.12‐1.23); for Hedonic Quality Identity, the score is significantly better for I2WE than BIKE-VG (*t*_87_=4; *P*<.001; *d*=0.99; 95% CI 0.37‐1.48) and for Total Attractiveness, the score is significantly better for I2WE than BIKE-VG (*t*_87_=2.83; *P*=.02; *d*=0.70; 95% CI 0.15‐1.69).

Scores on the physical activity enjoyment scale, the RTLX, the modified Borg scale, and the intensity of the session are provided in Table 3.

**Table 3.** Perceived enjoyment scores, workload, physical intensities.

Score	SWITCH	I2WE^a^	BIKE-VG^b^	*P* value	η²
Perceived Enjoyment (score)	60.48 (8.93)	60.71 (6.04)	52.72 (11.67)	**<.001**	**.15** ^**c**^
RTLX^d^ (score)	54.46 (18.54)	53.46 (14.28)	59.14 (16.36)	.34	.02
Mental demand (score)	62.39 (23.25)	63.16 (25.8)	75.00 (18.03)	.07	.06
Physical demand (score)	60.65 (23.47)	62.37 (26.65)	51.72 (29.74)	.25	.03
Temporal demand (score)	63.26 (24.24)	64.47 (23.79)	61.72 (23.19)	.90	.00
Effort (score)	56.96 (22.50)	57.63 (22.5)	57.07 (23.59)	.99	.00
Performance (score)	44.78 (24.47)	39.74 (16.52)	54.66 (25.46)	**.03**	**.08**
Frustration (score)	38.70 (31.7)	33.42 (23.11)	54.66 (27.48)	**.01**	**.11**
Modified Borg Scale (score)	3.83 (1.11)	4.37 (1.55)	3.45 (1.11)	**.03**	**.08**
Mean Intensity (percent of TMHR)^e^	68.01 (8.2)	69.64 (9.11)	63.41 (8.95)	**.02**	**.09**

a I2WE: Immersive and Interactive Wall Exergame

b BIKE-VG: Bike-based Video Game group

c Bold values are significant.

d RTLX: ‘‘Raw’’ Task Load Index

e TMHR: theoretical maximal heart rate

### Perceived Enjoyment

There was a significant group effect (F_2,87_=7.71, *P*<.001, η²=.15) for *Perceived Enjoyment*. Post Hoc analysis revealed a significant difference between I2WE and BIKE-VG (t_87_=3.63, *P*=.001, *d*=0.90, 95% CI 2.74‐13.23) and between SWITCH and BIKE-VG (t_87_=3.11, *P*=.01, *d*=0.87, 95% CI 1.82‐13.69).

### Session’s Workload

The session’s workload, evaluated by the RTLX, was correct for the three groups. The three dimensions related to the task (ie, mental demand, physical demand, and temporal demand) were balanced, while frustration was the lowest dimension, except for BIKE-VG, where the frustration was higher. There was a significant group effect (*F*_2,87_=5.30; *P*=.01; η²=.11), and the post hoc test revealed a significant difference between I2WE and BIKE-VG (*t*_87_=3.20; *P*=.01; *d*=0.79; 95% CI 5.41‐37.06).

There was also a significant group effect (*F*_2,87_=3.88; *P*=.03; η²=.08) for performance. The post hoc test indicated a significant difference between I2WE and BIKE-VG (*t_87_*=2.77; *P*=.02; *d*=0.68; 95% CI 2.09‐27.75).

### Physical Intensity

The objective physical intensity was moderated for the three groups, but there was a significant group effect (*F*_2,87_=4.22; *P*=.02; η²=.09) for mean intensity. The post hoc test showed a significant difference between I2WE and BIKE-VG (*t*_87_ = 2.86; *P*=.01; *d*=0.70; 95% CI 1.04‐11.43)

The intensity was perceived between very easy and easy for SWITCH (*M*=3.93) and BIKE-VG (*M*=3.45) and between easy and moderate for I2WE (*M*=4.37). There was a significant group effect for subjective intensity (*F*_2,87_=3.58; *P*=.03; η²=.08). I2WE was significantly greater than BIKE-VG (*t*_87_=2.64; *P*=.03; *d*=0.65;95% CI 0.09‐1.75).

### Want to Continue to Play Active Video Games

In all 3 groups, most participants wanted to continue: 84% (32 out of 38 for I2WE; 58% completely agreed, 26% agreed), and 65% for SWITCH (15 out of 23; 43% completely agreed, 22% agreed). However, these figures were lower for BIKE-VG with 59% (17 out of 29; 21% completely agreed, 38% agreed). A nonnegligible proportion disagreed (17%) or completely disagreed (14%).

## Discussion

### Principal Findings and Comparison With Previous Works

This study aimed to compare three AVG experiences (I2WE, SWITCH, BIKE-VG) on older adults. Concerning UX, the overall attractiveness, which is the total perceived value of the product based on the perception of its pragmatic and hedonic qualities, was positive for I2WE and SWITCH. Games-based programs could offer an enjoyable and stimulating exercise experience [4]. AVG provide instant visual and auditive feedback, motivational messages such as bonuses, or encouraging comments that make the activity entertaining and interactive [48]. However, the overall attractiveness was not positive for the three experiences, given that it was neutral for BIKE-VG, with a significant difference between I2WE and BIKE-VG. According to Lallemand and Gronier [49], the neutral zone indicates that the product fulfills its purpose without causing any negative impact. The difference between the two other groups may be explained by the combination of physical and cognitive stimulation, which is less natural for BIKE-VG than for I2WE and SWITCH: the physical task (ie, cycling) is not directly linked to the cognitive task (ie, video games). Exercises involving Thinking While Moving may be less instinctive and ecological. In contrast, I2WE and SWITCH involved Moving While Thinking tasks: the cognitive task was incorporated into the physical tasks. Moving While Thinking training may be easier, especially since this was the first experience with AVG for most participants in this study. Although there was no significant difference between the groups, this could explain why the BIKE-VG program, a Thinking While Moving program, had a high cognitive scale score on the workload scale (75/100) compared with the other two Moving While Thinking training programs, with 62.39/100 for the SWITCH group and 63.16/100 for the I2WE group. The difference in overall attractiveness may also be partially attributed to the variation in interaction on with other participants: BIKE-VG used single-player game, while SWITCH and I2WE involved multiplayer games.

Moreover, the controller may be the origin of this dissimilarity, reflected by differences in the Pragmatic Quality scores. Its evaluation represents users’ perceived ability to use products that allow them to manipulate the environment to accomplish their objectives. The Pragmatic Quality score was neutral for the three groups; however, the score was significantly lower for BIKE-VG than for I2WE and SWITCH groups. A button controller may be more complex to use than another controller (ballon controller or mixed button and gesture controller) and need more learning effort [50]. This may be creating an alteration of the experience. In their study, Pham and Theng [50] found that the older participants preferred to play games with fewer or no controlling devices. The Gesture Controller (Kinect), which was reported to require much physical movement, scored the highest in overall attractiveness, while participants gave negative feedback to the button controller, which requires fewer body movements. A difficulty in using the buttons can explain lower score for the BIKE-VG group and can, therefore, generate more frustration and worse performance than the body or mixed button and gesture controller. Indeed, the BIKE-VG group had a higher frustration score at NASA-TLX than the I2WE group. Moreover, it was the same for performance. However, the global workload score was correct for the three groups, with no significant difference between the three groups.

AVG promise to reduce loneliness, increase social connection, and foster positive attitudes toward others [18]. However, AVG may also provide opportunities for social engagement with peers and family members [48]. In this study, this is reflected by the positive score of the I2WE groups for the Hedonic Quality Identity score, which is the capacity to support a social function and communicate a specific user identity. This AVG provides a lot of communication and interaction between players. The BIKE-VG group, where the participants played alone (single-player), obtained a neutral score significantly lower from I2WE (multiplayer). Moreover, SWITCH and I2WE supported the need for stimulation with new, attractive, and stimulating content, features, and interaction styles, given that the two groups had positive scores at Hedonic Quality Stimulation. The score was neutral for BIKE-VG, but no significant difference existed between the groups. It is possible that the BIKE-VG session was too monotonous because it contained only one game, compared to SWITCH and I2WE, which included several games.

The intensity was moderated for the three groups, between 50% and 70% of the maximum heart rate, and almost high intensity for the I2WE and SWITCH groups. This is in line with the ACSM recommendations. Therefore, these three AVG could be used in a training program to improve physical and cognitive abilities. However, the physical intensity was significantly greater for I2WE than for BIKE-VG. The physical solicitation of I2WE may explain this difference: the whole-body multi-domain training and the freedom of movement offered by the system may increase intensity, as Martin-Niedecken showed with the ExerCube [9]. Moreover, Moving While Thinking training may increase intensity when two tasks are linked (there is no task prioritization). Subjectively, the intensity was considered very easy for SWITCH and BIKE-VG and easy to moderate for I2WE, with a significant difference between I2WE and BIKE-VG. The physical stimulation, which was intermittent for I2WE and SWITCH and continuous for BIKE-VG, may explain these differences.

As indicated in many studies [27,33,51,52] and the present work, the pleasure generated by AVG is high. However, it was significantly higher for I2WE and SWITCH than BIKE-VG. Some AVG create more enjoyment than others, depending on the features of the interfaces and the interactions between players. Indeed, as noted by Caserman et al [53], social interaction, one of the many quality criteria required for a game, plays an important role in enhancing enjoyment. Concerning the interface, Kim et al [35] showed that the level of interface embodiment influences the level of enjoyment on young adults (mean age =24.39). They compared three levels of interface embodiment (low, medium, and high), finding that the high level provided significantly more enjoyment than the other two.

The individual characteristics of participants may influence each user’s experience. In this study, no differences were found between age, BMI, physical activity level, gender, education level, and previous experience with active video games. Although most participants (82%-90% depending on the group) had never played active video games, players’ preferences and profiles may still affect UX, enjoyment, or physical intensity.

According to our knowledge, this study was the first to compare three AVG experiences in older adults. Thus, the comparison with the literature is thin. For example, studies often compare only 2 devices [54], or sometimes 3, but with sessions lasting a few minutes [26] that do not reflect a complete session.

### Conclusion

In light of the present study, Moving While Thinking training (eg, I2WE, SWITCH) appears promising for stimulating physical, cognitive, and dual-task capacities in older adults. An interventional approach is needed to compare the effects of these programs to Thinking While Moving programs (eg, BIKE-VG).

## Abbreviations

AVG: active video game
BIKE-VG: bike and video games
I2WE: immersive and interactive wall exergames
IRRID: International Registered Report Identifier
NASA-TLX: NASA-Task Load Index
PACES: Physical Activity Enjoyment Scale
QAPPA: Questionnaire d’Activité Physique pour les Personnes Âgées
RTLX: Raw Task Load Index
TMHR: Theoretical Maximum Heart Rate
UX: User Experience
